# A Web-Based Coping Intervention by and for Parents of Very Young Children With Type 1 Diabetes: User-Centered Design

**DOI:** 10.2196/diabetes.9926

**Published:** 2018-12-17

**Authors:** Tim Wysocki, Jessica Pierce, Cindy Caldwell, Karen Aroian, Louis Miller, Rebecca Farless, Ivy Hafezzadeh, Terri McAninch, Joyce M Lee

**Affiliations:** 1 Nemours Children's Specialty Care Center for Health Care Delivery Science Nemours Children's Health System Jacksonville, FL United States; 2 Nemours Children's Hospital Center for Health Care Delivery Science Nemours Children's Health System Orlando, FL United States; 3 College of Nursing University of Central Florida Orlando, FL United States; 4 eCity Interactive, Inc Philadelphia, PA United States; 5 Family Advisor Ormond Beach, FL United States; 6 Family Advisor Hayward, CA United States; 7 Nemours Foundation Department of Marketing and Communication Nemours Children's Health System Jacksonville, FL United States; 8 Child Health Evaluation Research Department of Pediatrics University of Michigan Ann Arbor, MI United States

**Keywords:** coping, mobile phone, parenting, social media, type 1 diabetes

## Abstract

**Background:**

Management of type 1 diabetes (T1D) among children aged <6 years is exceptionally challenging for parents and caregivers. Metabolic and psychosocial outcomes among very young children with T1D (YC-T1D) are tightly associated with their parents’ ability to meet these challenges. There is scant research testing interventions targeting these issues and few resources to equip health care providers with feasible and effective coping strategies for these parents. User-centered design (UCD) of a continuously accessible Web-based resource could be a mechanism for helping parents of YC-T1D cope more effectively with the complex challenges they face by providing them with information, solutions, and emotional support.

**Objective:**

The objectives of this paper are to (1) describe the application of UCD principles to the development of a Web-based coping intervention designed by and for parents of very young children (<6 years old) with T1D; (2) illustrate the use of crowdsourcing methods in obtaining the perspectives of parents, health care providers, and Web development professionals in designing and creating this resource; and (3) summarize the design of an ongoing randomized controlled trial (RCT) that is evaluating the effects of parental access to this resource on pertinent child and parent outcomes.

**Methods:**

This paper illustrates the application of UCD principles to create a Web-based coping resource designed by and for parents of YC-T1D. A Web-based Parent Crowd, a Health Care Provider Crowd, and a Focus Group of minority parents provided input throughout the design process. A formal usability testing session and design webinars yielded additional stakeholder input to further refine the end product.

**Results:**

This paper describes the completed website and the ongoing RCT to evaluate the effects of using this Web-based resource on pertinent parent and child outcomes.

**Conclusions:**

UCD principles and the targeted application of crowdsourcing methods provided the foundation for the development, construction, and evaluation of a continuously accessible, archived, user-responsive coping resource designed by and for parents of YC-T1D. The process described here could be a template for the development of similar resources for other special populations that are enduring specific medical or psychosocial distress. The ongoing RCT is the final step in the UCD process and is designed to validate its merits.

## Introduction

Type 1 diabetes (T1D) is increasing in prevalence among children aged <6 years [1-3]. Daily T1D care is immensely challenging for parents and caregivers, and the adequacy of parental coping is intertwined tightly with their children’s metabolic and psychosocial outcomes [4-14]. Yet, there are few resources that specifically target the unique needs of this population or that equip health care providers to offer feasible and effective coping strategies to these parents and caregivers [15,16].

The development, evaluation, and dissemination of digital health interventions for promoting healthy lifestyle and improved management of chronic medical conditions [17-23] are growing. These include websites [24-26], smartphone apps [27,28], and innovative devices [29] designed to assist people in achieving specific health goals. The development of these interventions is labor-intensive and costly. Hence, developers of these resources would be prudent to include targeted end users throughout the design process to ensure the utility and uptake of new interventions.

Many have advocated the application of user-centered design (UCD) in the development of digital health resources. Roberts et al [30] advocated for the application of design thinking in the area of innovations in health care management. Maher et al [31] described their process of developing a roadmap for bone marrow transplant patients built largely on input from patients who represented end users of this tool. LeRouge and Wickramasinghe [32] reviewed research applying UCD principles in the design and development of diabetes-related consumer health information technology initiatives and platforms. The authors concluded that few projects have verified the use of UCD principles throughout the entire life cycle from conceptualization to implementation of the end product. Devito-Dabbs et al [33] illustrated the merits of UCD in their development of a “Pocket Personal Assistant for Tracking Health” device for the promotion of self-management behaviors in lung transplant patients. The UCD principles that drove their design process were (1) Focus on Users and Tasks; (2) Measure Usability Empirically; and (3) Design and Test Usability Iteratively. The authors demonstrated how these UCD principles could guide the development of a wide range of digital health interventions. This paper illustrates how we applied these same UCD principles to the entire life cycle of our design and the development of a Web-based coping resource created by and for parents of very young children with T1D.

Extensive research shows that effective management of pediatric T1D requires substantial involvement of patients and family members, and outcomes are heavily dependent on how families accommodate the demands of T1D to their daily lives. This point is especially salient for parents of very young (<6 years old) children with T1D (YC-T1D) because YC-T1D lack the cognitive, behavioral, and emotional self-regulation skills that are prerequisites for T1D self-management. Hence, YC-T1D are prone to display difficulties adapting to the demands of care, as manifest in resistance to painful procedures, mealtime behavioral problems, anger over perceived differential treatment relative to peers or siblings, etc [4-16]. Parents of YC-T1D are overwhelmed, anxious, and prone to fatigue owing to their pervasive worry about their children, constant vigilance about their children’s blood glucose levels, and reluctance to place their children in the care of others [9,10]. Multifamily support groups that specifically target these parents encounter barriers such as the low incidence of T1D in very young children, frequent acute childhood illnesses, caregiving duties for other young children, interference with children’s early bedtimes, and hesitation about others caring for their children. While many centers offer general support groups for children and teens with T1D, they do not address the unique issues faced by parents of YC-T1D. Since this population of parents tends to be heavy internet and social media users [34], it seems plausible that they could benefit from a Web-based coping resource.

This paper describes the application of UCD principles [30-33,35] to the development of a Web-based coping intervention “by and for” parents of YC-T1D. This paper illustrates the application of UCD principles by engaging many parents of YC-T1D, health care providers, and experts in T1D medical and psychosocial care (JML, TW, and JP), qualitative research (KA), Web development (LM and CC), and usability testing (TM) in this initiative. The research team relied extensively on crowdsourcing methods to facilitate the UCD process. Crowdsourcing, a flexible Web-based activity [36] that has been applied to problems in diverse fields, comprises 4 elements [37]: (1) an organization that has a task it needs to be performed (eg, design an internet resource meeting parents’ specifications); (2) a community, or crowd, that contributes to meeting those specifications; (3) a Web-based environment that enables collaboration between the crowd and the organization; and (4) mutual benefit for the organization and the crowd (eg, better child health and quality of life, less family distress). Crowdsourcing methods enabled the researchers to efficiently capture the perspectives of parenting roles and challenges from a relatively large and diverse group of parents of YC-T1D, ensuring that the design process was consistent with the “by parents, for parents” approach. With extensive stakeholder engagement, the researchers developed a continuously accessible resource that provides credible information and social support and offers searchable content that evolves in response to ongoing needs and preferences of the user group. The development of the Web-based resource is now complete, and recruitment of participants for a randomized controlled trial (RCT) has begun.

## Methods

**Figure 1:**
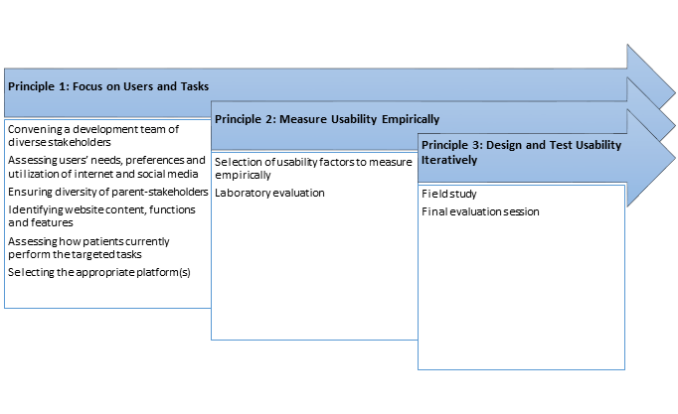
User-centered design principles applied in this project.

Figure 1 depicts the multistep, UCD process employed here, followed by more detailed treatment of those elements.

### Principle 1: Focus on Users and Tasks

#### Convening a Development Team of Diverse Stakeholders

Parents of YC-T1D and health care providers served multiple roles on the development team, with a corresponding range of engagement methods. Constitution of the development team began during the earliest stages of the grant application that supports this work. Using a variety of recruitment approaches (nominations by T1D professionals, referrals from diabetes advocacy groups, and internet advertising), 5 parents of YC-T1D agreed to serve as Family Advisors to the research team in preparing the project plan and securing funding for the project. They met with the first 2 authors approximately monthly during the preparation of the project plan to provide stakeholder input. The process of securing funding for the project required about 9 months, during which time there was an interlude in any activity involving the Family Advisors. Once funding was secured for the project, the team recruited a Web-based community (“crowd”) of many parents of YC-T1D to guide the planning of the website and reconstituted the group of Family Advisors. Of the original Family Advisors, 3 committed to continuing in that role, and the researchers recruited 3 additional Family Advisors, comprising a team of 6 that would advise the researchers throughout the remainder of the design and development of the Web-based resource, as well as the implementation of the RCT to follow.

Based on the Family Advisor input, the team began the design process with a systematic effort to characterize the parents’ perspectives of challenges their families faced in meeting the unique needs of YC-T1D, while also addressing their other personal, marital, family, and vocational priorities. The intent of this step was to provide a broad perspective of the psychological landscape faced by these families to guide future iterative interaction with a larger Web-based parent community to progressively refine and validate this framework. Using recruitment methods similar to those employed in recruiting the Family Advisors, we then assembled a Web-based Parent Crowd, who were interested in assisting the researchers in designing and building the Web-based resource. Parents were eligible if they were parents or legal caregivers of a child who was diagnosed with T1D before the age of 6 years and was aged <10 years at the time of recruitment. Relying on both direct contact with parents of YC-T1D at the host institution and contact through a variety of resources comprising the “Diabetes Online Community” [38], a group of 170 parents enrolled as Parent Crowd members, of whom 153 participated actively in the design of the Web-based resource as described below. Although it might have been valuable to characterize these parents in terms of the type and quality of T1D care received by their children, the researchers did not attempt to collect that type of information. The parents’ children received care at numerous different centers, and self-report by parents is probably not the most accurate way to characterize a given center’s clinical resources and practices. The depth and quality of parents’ responses to the researchers’ Web-based qualitative questions suggest a sample of parents that were receptive to T1D technology and highly engaged in their children’s care.

Conference calls between the Family Advisors and the researchers occurred every 2-4 weeks throughout the project phase, contributing a wide range of input into various project decisions and plans and ensuring that those plans adequately reflect broader Parent Crowd input. For example, Family Advisors reviewed and edited the instructions for several Parent Crowd tasks, detailed below, to ensure they were easily comprehensible; reviewed the list of parent-generated and health care professional-generated articles written for the website and suggested additional articles to be written; and reviewed and confirmed that changes made to the website following usability testing (see Principle 2 below) were consistent with the preferences specified by Parent Crowd members. Parent Crowd members strongly recommended that the website should be structured so that parents of newly diagnosed children would not be overwhelmed by the magnitude and scope of many challenges that they, their children, and their other family members would now face. As a result of this input, parents who log in to the website for the first time receive a prompt asking whether their child is very recently diagnosed. Those who respond affirmatively are directed to content that was specifically selected by the Parent Crowd and research team as being most appropriate for these parents. This content included opportunities for basic education about T1D, diagnosis stories submitted by Parent Crowd participants, and a variety of articles about getting through the early weeks and months after the diagnosis.

**Open-ended questions distributed to the Parent Crowd via Yammer for written replies.**

1. In what ways has your life changed since your child was diagnosed with type 1 diabetes?
2. What challenges are you facing in managing your child’s diabetes? If your child is currently 6 or older, please answer this question about the challenges you experienced when he/she was 5 or younger.
3. What do you do now that helps you cope with the challenges you described in Question 2? If your child is currently 6 or older, what did you do to cope when he/she was 5 or younger?
4. How does/did being a parent of a very young child with diabetes affect your relationships with others?
5. In what ways has your child’s life changed since he/she was diagnosed with T1D?
6. How does your child’s behavior or temperament affect your ability to take care of diabetes? Please remember to answer this question about your experience when your child was 5 or younger.
7. How has taking care of your child’s diabetes affected your other children, if you have any? Please remember to answer this question about your experience when your child was 5 or younger.
8. How do you fit diabetes care into your daily family life? If your child is currently 6 or older, how did you fit diabetes care into your daily family life when he/she was 5 or younger?
9. How have you fit your child’s diabetes care into special occasions (holidays, birthdays, travel)? If your child is currently 6 or older, how did you fit diabetes care into special occasions when he/she was 5 or younger?
10. What could your diabetes care team do to be more helpful to you in caring for your child? If your child is currently 6 or older, please answer this question about what your health care team could have done when your child was younger than 6.
11. Looking back, is there some aspect of caring for your child that you could have been better prepared for?
12. Knowing what you know now, what is the most important advice you would give to a parent whose young child was just diagnosed?
13. What advice or information about treating young children, toddlers, and infants with T1D would you give to your child’s doctor or health care team? What would you like them to know?
14. In what ways, if any, has raising a young child with diabetes been a positive experience for you?
15. In what ways, if any, has diabetes been a positive experience for your young child?
16. What else would you like us to know about your experience raising a young child with T1D that wasn’t address in the questions you have already answered?

17-19. Intimacy questions (Sent via email rather than posted on Yammer given private nature of content)

Are you married or living with a partner?

If YES:

- How do you and your spouse or partner divide responsibility for your child’s diabetes care? How acceptable is this arrangement to each of you?
- In what ways has your child’s diabetes affected the emotional intimacy or closeness of your relationship with your spouse or partner?
- In what ways has your child’s diabetes affected the physical intimacy or closeness of your relationship with your spouse or partner?

If NO:

- How successful have you been in finding others who you trust to care for your child with T1D?
- In what ways do you do things just for yourself, to give yourself a break?
- In what ways has your child’s diabetes affected your life in the areas of dating and romance?

#### Assessing Users’ Needs, Preferences, and Utilization of the Internet and Social Media

Based on the Family Advisor input, the researchers’ knowledge of the pertinent research evidence base, and with consultation from a qualitative research expert (KA), the researchers developed 16 a priori open-ended questions for distributing among the Parent Crowd through a private social network, Yammer. The questions sought to characterize the challenges faced while parenting YC-T1D in terms of its impact on YC-T1D, parents, marital and family issues, extrafamilial social relationships, workplace and career issues, and interactions with the health care community. Three additional open-ended questions were developed as the team gained experience with this subject matter, addressing marital intimacy, workplace issues, and relationships with health care providers in more depth. For the 19 open-ended questions, shown in Textbox 1, participants responded by entering written replies that were available to all Parent Crowd members, providing opportunities for interactions among Parent Crowd members about their perspectives and experiences. The researchers distributed 15 other polls and surveys designed to characterize their use of the internet in general and specific to T1D, use of social media as a means of obtaining T1D information and support, use of other sources of T1D information, and experiences in multifamily T1D support groups. These efforts yielded a Social Ecological Model (Figure 1) that provided a taxonomy for organizing the functional domains that should be addressed by the planned Web-based resource [9].

Certain proposals raised by the Parent Crowd members, such as broadening intended website users to include parents of older children with T1D and providing T1D educational games for children on the website, indicated that the Parent Crowd’s work could proceed most efficiently if it could become more focused. The Family Advisors and the investigators proposed that the group should develop a formal Vision, Mission, and Operating Principles document to ensure consistency of the group’s purposes and strategy and to more clearly define the nature of the end product that should result from this work. Over several iterations, the participants prepared successive drafts of a Vision, Mission, and Operating Principles document. The final document, ratified by a Parent Crowd vote, is shown in Textbox 2, and it is posted prominently on the completed website.

**Vision, Mission, and Operating Principles document.**

**Vision**

Our vision is to provide a comprehensive internet resource designed by parents for parents of infants, toddlers, and preschoolers with type 1 diabetes (T1D).

**Mission**

The dual mission of this website is as follows:

- To ensure parents and caregivers have the information, resources, and support they need to promote the health and well-being of their child(ren) with T1D
- To provide parents and caregivers of children with T1D the information, support, and resources that they can use to enhance their own physical and mental health and well-being.

**Operating Principles**

1. The website content and features will be managed by the Website Committee, comprising the Researchers and Family Advisors. In all of its activities, the committee will ensure that the website is developed “by parents for parents.”
2. Anyone can submit content or suggestions to the Website Committee for possible posting on the website.
3. To the extent possible, the website will offer a “one-stop” resource for parents (or other caregivers) of infants, toddlers, and preschoolers with T1D.
4. The website will offer both informational and social media resources.
5. The website will help parents and caregivers connect with others who share similar circumstances or concerns.
6. The website will provide links to reputable and helpful external resources (eg, websites, books, and agencies), its content will be kept current and will grow in response to users’ needs, and all medical information on the website will be accurate and credible.
7. Users will respect each other by being polite and accepting others’ diverse experiences and opinions.
8. The website will enable parents and caregivers to set their own preferences for safeguarding their privacy and confidentiality.
9. The website content will be accessible and useful to parents, providing a wide range of reading and internet use skills.
10. Although the informational content on the website will be in English, it will enable people who prefer other languages to connect with each other.

**Table 1:** Demographic characteristics of young children with T1D whose parents who took part in specific website development components.

Characteristics		Parent crowd group (n=153)		Diversity focus group (n=13)		Usability test participants (n=10)	
		n	Mean (SD)	n	Mean (SD)	n	Mean (SD)
**Child characteristics**							
	Age (years)	139 (90.8)	5.50 (2.00)	8 (62)	4.75 (1.39)	7 (70)	5.05 (2.61)
	Age at diagnosis (years)	138 (90.2)	2.63 (1.45)	8 (62)	2.63 (1.30)	7 (70)	2.87 (1.37)
	Duration of type 1 diabetes (years)	138 (90.2)	2.43 (1.97)	8 (62)	1.63 (1.19)	7 (70)	2.17 (2.89)
	Most recent hemoglobin A_1c_ (%)	134 (87.6)	7.69 (0.92)	7 (54)	8.43 (0.09)	6 (60)	8.48 (1.79)

**Table 2:** Demographic characteristics of young children with T1D whose parents who took part in specific website development components.

Characteristics		Parent crowd group (n=153)	Diversity focus group (n=13)	Usability test participants (n=10)
**Gender, n (%)**		**N=137**	**N=8**	**N=7**
	Male	65 (47.4)	3 (37.5)	4 (57.1)
	Female	72 (52.5)	5 (62.5)	3 (42.9)
**Race, n (%)**		**N=138**	**N=8**	**N=7**
	Caucasian	123 (88.5)	2 (25.0)	5 (71.4)
	African American	2 (1.4)	5 (62.5)	1 (14.3)
	Other or multiple	13 (9.4)	1 (12.5)	1 (14.3)
**Ethnicity, n (%)**		**N=133**	**N=8**	**N=7**
	Hispanic	9 (6.8)	3 (37.5)	2 (28.6)
	Non-Hispanic	124 (93.2)	5 (62.5)	5 (71.4)
**Insulin regimen, n (%)**		**N=136**	**N=8**	**N=7**
	Insulin pump	94 (69.1)	3 (34.5)	1 (14)
	Multiple daily injections	38 (27.9)	5 (62.5)	4 (57)
	Conventional or sliding scale	4 (2.9)	0 (0)	2 (29)
**Use of continuous glucose monitor, n (%)**		**N=138**	**N=7**	**N=7**
	Yes	96 (69.6)	5 (62.5)	4 (57.1)
	No	42 (30.4)	2 (29)	3 (57.1)

#### Ensuring Diversity of Parent Stakeholders

Since the Parent Crowd members were disproportionately Caucasian, married, and college educated and had above average in household income, the research team constituted a 13-member Diversity Focus Group, essentially doubling minority representation on the project team, ensuring that the design process reflected the perspectives of racially, ethnically, and economically diverse parents. The Diversity Focus Group participated through videoconference from 1 of the 3 locations. The researchers condensed the 19 open-ended questions previously distributed to the Parent Crowd to 6 summative questions posed to the Diversity Focus Group. The Focus Group results supported the working Social Ecological Model and confirmed the previous findings from the Parent Crowd. Some new examples of specific issues emerged, but the research team concluded that the perspectives of the Diversity Focus Group were very similar to those of the Parent Crowd.

Tables 1-3 summarize the demographic characteristics of the Parent crowd, participants in the Diversity Focus Group, participants in a usability testing session described below, and their YC-T1D. Usability testing participants were more diverse than the Parent Crowd members, particularly in the inclusion of 50% male caregivers within that sample.

**Table 3:** Demographic characteristics of parent participants who took part in specific website development components.

Characteristics		Parent crowd group (n=153)	Diversity focus group (n=13)	Usability test participants (n=10)
Parent age (years), mean (SD)		36.34 (5.6)	34.4 (7.0)	35.5 (5.2)
**Relationship with child, n (%)^a^**				
	Biological mother	129 (84.6)	8 (61.5)	6 (60.0)
	Biological father	22 (14)	3 (23.1)	3 (30.0)
	Other	2 (1)	2 (15.4)	1 (10.0)
**Education, n (%)^a^**				
	HS diploma	12 (7.8)	4 (33.3)	0 (0)
	Some college or technical school	41 (26.8)	5 (41.7)	10 (100.0)
	Bachelor’s degree	54 (35.3)	1 (8.3)	0 (0)
	Advanced degree	45 (29.4)	2 (16.7)	0 (0)
**Occupation, n (%)^a^**				
	Not employed outside home	44 (28.8)	4 (33.3)	3 (30.0)
	Operational or technical level	29 (19.0)	4 (33.3)	0 (0.0)
	Managerial level	48 (31.4)	3 (25.0)	4 (40.0)
	Professional level	26 (17.0)	1 (8.3)	3 (30.0)
**Household annual income, n (%)^a^**				
	<US $50K	32 (20.9)	5 (41.7)	2 (20.0)
	US $51K-US $100K	63 (41.2)	5 (41.7)	8 (80.0)
	US $101K-US $150K	34 (22.2)	2 (16.7)	0 (0.0)
	>US $150K	17 (11.1)	0 (0.0)	0 (0.0)
**General internet use, n (%)^a^**				
	Daily	103 (67.3)	4 (33.3)	7 (70.0)
	Often	23 (15.0)	2 (16.7)	1 (10.0)
	Sometimes	19 (12.4)	4 (33.3)	2 (20.0)
	Never	3 (2.0)	1 (8.3)	0 (0)
**Type 1 diabetes-related internet use, n (%)^a^**				
	Daily	60 (29.2)	2 (16.7)	5 (50.0)
	Often	45 (29.4)	1 (8.3)	1 (10.0)
	Sometimes	27 (17.6)	5 (41.7)	3 (30.0)
	Never	11 (7.2)	2 (16.7)	1 (10.0)

^a^Percentages were calculated using a denominator consisting of the number of parents reporting on the dimension of interest. Hence, the denominators varied among the different demographic characteristics.

#### Identifying Website Content, Functions, and Features

**Figure 2:**
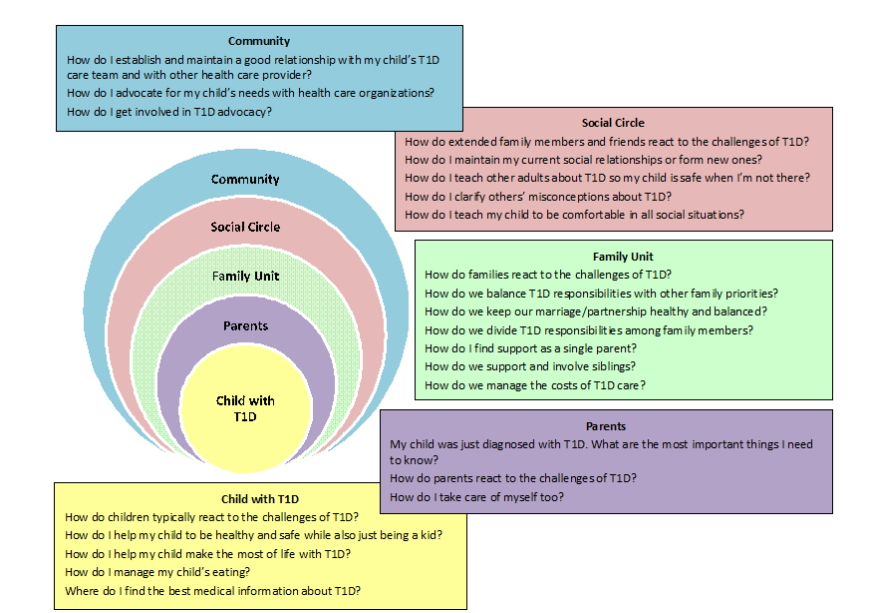
A social ecological taxonomy of influences on family management of type 1 diabetes (T1D) in very young children.

As delineated in Textbox 1, the Parent Crowd’s responses to the 19 open-ended questions yielded voluminous information about the many ways in which raising a YC-T1D impacted every corner of their lives. The Parent Crowd then assisted the research team in condensing the perspectives they contributed into a set of 23 parenting challenges that the Web-based resource should help users address. Specifically, an initial draft of these issues was posted to the Parent Crowd members who were asked to rate the clarity of each challenge and to provide feedback about each challenge. These challenges, shown in Figure 2 as questions, served as a guide to developing the website’s features, functions, and information architecture. The team also used these challenges to generate topics for parent-authored and health care professional-authored articles for the website. Furthermore, Parent Crowd members submitted “Questions for the Experts” that were compiled into topics, and then the researchers recruited specific health care professionals to contribute additional articles on those topics.

#### Assessing How Patients Currently Perform the Targeted Tasks

A very high proportion of the Parent Crowd indicated weekly or more frequent use of the internet or social media for both general and T1D-specific purposes. However, the Parent Crowd members expressed considerable dissatisfaction with the availability of credible information on the internet that is specific to YC-T1D. They also reported receiving limited T1D-specific, in-person support because of friends and family members not being able to understand the complexities of raising YC-T1D. Hence, the Parent Crowd members tended to describe themselves as socially isolated, as having relatively little contact or support from other parents in their situation and as being largely alone in finding solutions to T1D challenges. Based on this input, the research team reasoned that the needs of parents of YC-T1D could be well served if they had continuous access to a Web-based resource that provides them with the information and support they need to optimize their children’s health and development, while also preserving their own health and well-being.

#### Selecting the Appropriate Platform(s)

The development team elected to develop the Web-based resource using responsive Web design so it could be accessible by the full range of devices and screen sizes, including smartphones, desktop and laptop personal computers, notebooks, and tablet devices.

### Principle 2: Measure Usability Empirically

#### Selection of Usability Factors to Measure Empirically

Since 94% of 18-29 year olds and 89% of 30-49 year olds use smartphones [39], the research team decided to focus its usability testing work on that platform. Other reasons for deciding to focus first on the mobile phone platform rather than on the personal computer or notebook platforms were that the Web developers asserted that it is easier and less expensive to “scale up” the mobile phone platform rather than to “scale down” other platforms, that the project lacked the funds to conduct usability testing for all 3 platforms, and that the mobile phone platform was more likely to reveal navigation problems than the other 2 platforms. An expert in user-centered website design guided the team’s specification of activities for parents to perform on the website. The team assessed participants’ perspectives of the nature and purposes of the website and ease of navigation for common tasks and the degree to which the architecture and features were intuitive.

#### Laboratory Evaluation

The formal usability testing session targeted the assessment of these activities by parents of YC-T1D without prior knowledge of the team’s development of this resource. With an expert in usability testing, the team compiled a 46-item structured agenda (Multimedia Appendix 1) for the testing sessions. The team completed 1-hour individual usability testing sessions with 10 parents over 2 days at a marketing research firm. Participants used their own mobile phones to log in to a website prototype and then completed the usability testing session. After a brief orientation, the facilitator placed the parent’s mobile phone in a frame that held it stationary. A video camcorder placed above the phone recorded participants’ actions in response to the instructions. Participants were asked to describe aloud their reasoning as they navigated through these tasks. Other members of the research team observed the session through a one-way vision screen and could prompt the facilitator to ask follow-up questions. Each parent received US $125 compensation for their efforts.

Each session began with ascertaining participants’ general and T1D-specific life circumstances, asked individuals to complete various tasks, and concluded with an overall evaluation of the website’s clarity and ease of use, as well as suggested changes to the website. The tasks that each individual was asked to perform included the following: carrying out instructions related to logging into the website; navigating to several different components of the website; reading and reacting to several different articles on the website; after exploring the website, inferring the website’s intended users and objectives; demonstrating how to submit an article, photo, or video clip for posting on the website; returning to the home page from varied locations; and using the website “Search” function.

Each session was videorecorded and audiorecorded for subsequent analysis. Based on the usability testing results, the team incorporated 26 design improvements to the final website appearance, structure, and functions. These included the following: changing the icon signifying the “menu” function to a more intuitive icon; making navigation to the home page more salient; reducing wordiness and font size to limit the need for scrolling; eliminating the prompt for “newly diagnosed” after each user’s first log-in; reducing the size of parent quotes on home page as it occupied too much space; emphasizing the “Contact Us” link by moving the tab to the home page menu bar; making article overviews as brief as possible; and other similar changes.

### Principle 3: Design and Test Usability Iteratively

#### Field Study

As the website design proceeded, the research team delivered 3 webinars over about 3 months to the Parent Crowd to collect and integrate their feedback on the website structure, features, and content. The content and topics of the 3 sequential webinars provided the following: Webinar 1, review of the site map and wireframes; Webinar 2, review of the home page and article page; and Webinar 3, a tour of the completed website. Parents could access the webinars in real time (permitting the submission of written comments or questions to the presenter) or through a recording of the session. After each webinar, the Parent Crowd members responded to open-ended questions, and the responses were integrated into the working prototype. These sessions also confirmed that the website development was congruent with the Parent Crowd’s specifications and with the Website Vision, Mission, and Operating Principles, shown in Textbox 2. One parent expressed impatience with the duration of the design and development process, which required about 21 months rather than the projected upper limit of 18 months.

#### Final Evaluation Session

The final webinar demonstrated the completed website to Parent Crowd members who participated in either the live or recorded session at their convenience. The purpose of the webinar was to notify parents of the completion of the website, to invite them to register as users, and to request their input after exposure to the website. About 2 weeks after the webinar, the researchers distributed 9 pertinent open-ended, qualitative questions to the parents who had used the website. Transcripts of the verbatim responses were coded by trained members of the research team and interpreted by the investigators in consultation with an expert qualitative researcher. Parents’ responses confirmed that the website met their expectations; was user-friendly and engaging, contained appropriate content; was positive and encouraging in tone; and was free of difficult, confusing, or tangential information. The parents did not identify any substantial flaws in the website design or functionality.

Now that the website is functional, the research team has begun recruiting eligible parents to enroll in an RCT comparing usual care for T1D with and without access to the website. The RCT will enroll parents of patients from Nemours Children’s Health System by direct contact, as well as eligible parents of YC-T1D who receive care elsewhere, using T1D-focused social media groups, websites, and blogs. Regardless of the recruitment method, parents’ informed consent and participation in the RCT will occur solely over the Web. Outcome measures will include indices of parental and child outcomes measured at 0, 6, and 12 months. Parental outcomes include measures of their adjustment for managing their children’s T1D, treatment adherence, quality of life, psychiatric symptoms, social support, parenting self-efficacy, T1D family routines, fear of hypoglycemia, and benefit finding. Child outcome measures include hemoglobin A_1c_ and general and T1D-specific behavior problems. Members of the Parent Crowd who guided the creation of this resource can continue using the website during the RCT, but they will not otherwise participate in the research procedures.

## Results

The research team demonstrated serious and continuing engagement of key stakeholders through a Web-based community of 153 parents of YC-T1D supplemented by a Diversity Focus Group comprising 13 parents representing racial and ethnic minorities, as well as the involvement of health care professionals specializing in T1D and experts in qualitative research, Web development, and usability testing. The consistent involvement of 6 Family Advisors, who were also members of the Parent Crowd, ensured that design decisions reflected the parents’ perspectives and preferences. This work thoroughly characterized the pervasive challenges faced by these parents in their daily lives and yielded a taxonomy based on a socioecological model that drove the design of the structure, content, and functionality of a Web-based coping resource designed by and for these parents.

The collaborative adoption of a Website Mission, Vision, and Operating Principles document clarified the goals of the design and development phase of this initiative. Results of 15 polls and surveys distributed to the Parent Crowd characterized the internet access and utilization habits of this population and identified specific website content and features that parents nominated as being potentially helpful. For example, the Parent Crowd members ranked their 5 favorite T1D Web-based resources and their preferences for the website’s social networking platform (ie, a built-in platform vs a Facebook group). A structured process for naming the website resulted in the selection of “The New Normal” as the main website name, “The New Normal: A Community of Parents of Young Children with Type 1 Diabetes” as the home page by-line, and “TheNewNormalT1D.com” as the domain name. Finally, formal user testing and periodic webinar demonstrations of design progress further engaged the Parent Crowd in the website design process. This work yielded a functioning, private website that the researchers are now evaluating formally. All users must be authenticated to access the website with secure credentials provided by site administrators. The site is also hosted on enterprise-level cloud hosting that includes a firewall to protect against website hacks and attacks. The site has recurring backups to ensure data security.

**Figure 3:**
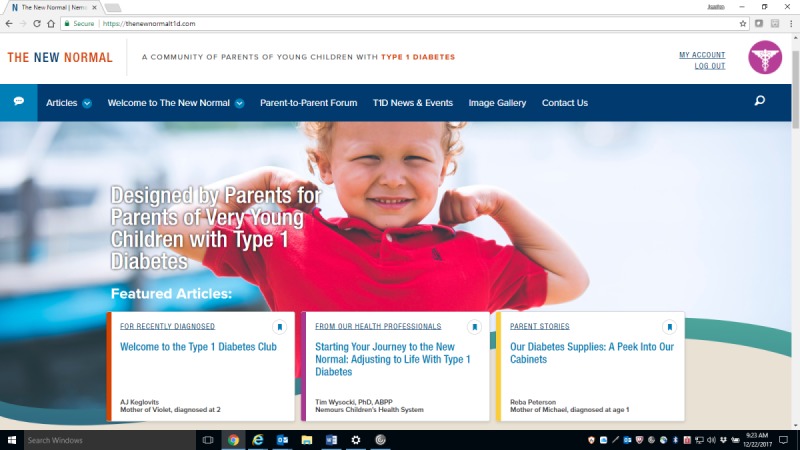
Screenshot of the website homepage home page with a menu bar depicting key functions and featured articles. Source: www.thenewnormalt1d.com.

The home page for The New Normal website, shown in Figure 3, demonstrates the functions, history, and development of the website; guidance on using the website’s features; articles written by parents or health professionals on topics suggested by parents; links to T1D-related news articles; and the Parent-to-Parent Forum, a private social media platform enabling parents to interact around topics of shared interest or discuss website articles. Other features include parent-contributed diagnosis stories and open letters to other parents, a photo gallery, a glossary of T1D technical terms, and a “Contact Us” utility that provides users with a variety of options such as offering the suggested content for articles, submitting news items, or reporting potentially erroneous statements that appear on the website. When a parent first accesses the website, an alert appears asking users if one or more of their children were recently diagnosed with T1D. If the parent clicks on “Yes,” links appear to content suitable to parents who are new to this role, including opportunities for learning or reviewing the fundamentals of T1D care, articles that describe typical reactions and adjustment to this experience, and articles that provide alternative methods for parent and child coping with the new diagnosis.

The Parent Crowd, in accord with the team’s professional Web development partners, advocated for continuously evolving content such that the website could be responsive to parent users’ needs; fresh and engaging to invite users to return repeatedly; and relevant to current developments in diabetes research, treatment, and health care policy. Consequently, the researchers designed the site to enable such features as archiving of discussion threads on the Parent-to-Parent Forum, multiple mechanisms for parent users to offer suggestions for new article topics, regular refreshing of news articles highlighted on the site, and periodic refreshing of photo images displayed on the site. The researchers regularly recruit professionals to contribute articles for the website on their areas of expertise and parents to write articles on special issues, such as one mother’s efforts to obtain a dog for her son trained to detect his hypoglycemic episodes. Articles or other website features that are not visited frequently will be removed, edited, or replaced.

The website utilizes the Woopra platform to track, compile, and analyze users’ patterns of website use. These data can be viewed on aggregated, subgroup, or individual user levels to identify pages relative to the frequency of use, frequency of return visits, and compilation of comments about article content. Monitoring and analysis of these data in the RCT will permit a careful understanding of who does and does not use the website, what users are most attracted to access, and what kinds of content attract users to revisit the website.

All website content is searchable using tags drawn from parents’ responses to the initial open-ended questions. The Parent Crowd expressed that the website should include strong safeguards to ensure that only medically accurate information appeared on the site. Assurance of medical credibility occurs during the editorial review of articles that parents or health professionals submit. Health professionals who are invited to contribute articles for the website are recognized experts in their respective disciplines. Articles submitted by either parent or professional authors are vetted, edited, and screened for scientific and medical credibility before posting on the website. The website manager, along with the website clinical directors, who are both pediatric psychologists with extensive T1D experience, conduct an initial review and then determine whether secondary review by the website medical advisor (JML) or another appropriate professional is needed. Additionally, the website includes a mechanism for parent users to report questionable content to the research team. Postings on the Parent-to-Parent Forum carry the highest risk of containing misleading or incorrect information, but several processes may prevent or reduce the appearance of inappropriate information on the website and ensure the prompt editing or removal of inappropriate content that is posted. These include parental self-policing, daily monitoring by the website manager and clinical directors, the Vision, Mission, and Operating Principles document that was adopted by the Parent Crowd and easily accessible on the forum, and the fact that this is a closed Web-based community.

## Discussion

This paper describes the application of principles of UCD to the crowdsourced creation of a Web-based coping resource developed by and for parents of YC-T1D. The design and development process sought extensive input from a Web-based community of parents that thoroughly characterized many challenges faced by these parents in seeking to preserve and optimize their children’s health, well-being, and overall growth and development. The preliminary qualitative work laid the foundations for collaborative specification of the structure, features, and content of the website and included the drafting, refinement, and adoption of a Vision, Mission, and Operating Principles document. Formal usability testing and periodic demonstrations of website design progress ensured that the website structure, features, and content embodied the Parent Crowd’s aspirations and preferences. The website is an organic resource that will continue to expand as parents contribute new articles, health care professionals contribute articles on topics requested by parents, and the use of the Parent-to-Parent Forum evolves over time.

An RCT is evaluating the potential benefits of website use in terms of the effects on child and parent outcomes. Unlike other interventions in which there is a specified number of sessions, doses, etc, there is no specified “end” to the use of the website, as it will be continuously updated. Thus, the research team also developed a website sustainability plan that includes a variety of initiatives designed to preserve the availability of this resource, including the submission of follow-up grant applications, solicitation of in-kind support for website hosting and maintenance from the host organization, cultivation of relationships with diabetes advocacy organizations that could assume responsibility for the website, and seeking philanthropic support from potential donors or corporate sponsors. The website could easily be the “go-to” resource for all families with children aged <6 years having a diagnosis of T1D. It was designed to provide a one-stop source for accurate information, practical help, and mutual support for these parents, essentially constituting a continuously accessible, highly specialized diabetes support group in cyberspace. This study provides a template for developing Web-based resources targeting other life challenges such as medical conditions, traumatic injuries, major changes in life circumstances, or other stressful life events. In addition to its applications to the website development, clinical researchers could apply the basic approach employed here in the development and evaluation of other digital health apps for smartphones, desktop personal computers, or tablet platforms.

Although the UCD process capitalized on the input of 4 groups of potential users (ie, Family Advisors, Parent Crowd, Diversity Focus Group, and Usability Testing Participants), there are several limitations to the study. Compared with traditional qualitative research, the crowdsourcing data collection method engaged a larger, geographically diverse sample to obtain a holistic perspective of the complex challenges faced by parents of YC-T1D. However, the Parent Crowd was not so diverse in terms of race, ethnicity, or socioeconomic status, and the website design should anticipate the needs and preferences of users with varied characteristics. Although the Parent Crowd had limited racial or ethnic diversity, our Focus Group participants consistently confirmed the data obtained previously from the larger Parent Crowd sample. Additionally, the majority of parent participants had YC-T1D who used insulin pumps and continuous glucose monitors and also had a lower-than-average mean hemoglobin A_1c_ [40]. This limitation raises concerns about the generalizability of findings across parents who have YC-T1D and are prescribed more conventional, less-intensive insulin regimens. However, a caveat that applies to UCD principles, as well as developing Web-based interventions for health, is that some populations may be more interested in and able to benefit from participating than others. Perrin and Duggan [41] recently reported the results of a Pew Research Center study on Americans’ internet use from 2000 to 2015. The internet use had essentially reached saturation among younger, more educated, more affluent people by 2015, while internet access and use was lower, but increasing slowly among older, less-educated people from lower socioeconomic strata. Some subpopulations, thus, continue to be “digital have-nots” who lack access to the internet. A certain level of fluency is also required, both in terms of comfort with technology and average to above average command of written language. The final limitation is that about one-third of participants had children who were aged between 6 and 10 years and were asked to report on their experiences before their child turned 6. For these parents, data were retrospective, and this might have introduced response bias. Nonetheless, parents of younger children, as well as the Family Advisors, often expressed that perspectives of parents whose children with T1D who were then aged >6 years was critical to the optimal design of the website.

UCD could play an important role in the ongoing development of such technologies as closed-loop insulin delivery systems [42], flash glucose monitoring [43], and cloud-based sharing of real-time blood glucose data [44]. These inventors and companies are not obligated to report on their engagement of key stakeholders from the beginning and throughout the completion of the design process and may choose to protect that information as proprietary. However, there would appear to be significant potential advantages to employing UCD principles, by ensuring that user perspectives precede, rather than follow, the design of the product in question. The engagement of stakeholders as key partners in the design of health interventions may slow the development process, but it may also yield interventions that are attractive and acceptable to end users, which they actually use and that achieve desired improvements in health and well-being.

## Appendix

Multimedia Appendix 1 Usability Testing Script.

## Abbreviations

RCT: randomized controlled trial
T1D: type 1 diabetes
UCD: user-centered design
YC-T1D: young children with T1D
