# Supplementary material for: A Web-Based Coping Intervention by and for Parents of Very Young Children With Type 1 Diabetes: User-Centered Design
Source: JMIR Diabetes. 2018 Dec 17;3(4):e16. doi: 10.2196/diabetes.9926 (PMC6307695; doi:10.2196/diabetes.9926)
Supplement: Multimedia Appendix 1 [file diabetes_v3i4e16_app1.pdf]

T1D Website Usability Test – MOBILE
A Crowdsourced Social Media Portal for Parents of Very Young Children with Type 1 Diabetes
"The New Normal: A Community of Parents of Young Children with Type 1 Diabetes"

August 3-4, 2017
Schlesinger Associates, Orlando (Maitland)

Prepared by: Terri McAninch, CUA, CXA
Design Services | Nemours Center for Health Delivery Innovation

Introduction

Thanks for taking the time to participate in this interview. I'm Terri McAninch, and I work with teams to help make their web sites easier to use. Today, you're going to help me evaluate a website for parents of young children with type 1 diabetes.

Specifics about the Process

- During the next hour, we'll talk about your experiences related to your child's diabetes. Then I'll ask you to use the website on your phone. If possible, please don't accept any calls or texts during our interview unless it's an emergency.

- As you use the website today, I'd like you to please "think aloud" as you look at the screens and tell me what's going through your mind, kind of like a running commentary. This will help me better understand what you're thinking.

- There are no wrong answers. We're evaluating the website design, NOT you. This is not an evaluation of your abilities. My goal is to get your feedback so we can make improvements to the website.

- I encourage you to be completely honest with your feedback and suggestions! I do work for the same organization as the researchers whose website we'll be looking at today, but I am not part of the project team, so you can't hurt my feelings.

- As you have already learned, there are a few of my colleagues observing this session to help me take notes, and we are taping the interview to help me when I do the final analysis. But all of the information you provide is kept strictly confidential and your name will not be associated with any of the records created for this study. I just want to make sure again that you are OK with that.

- Do you have any questions or concerns before we get started?

BACKGROUND QUESTIONS (5 minutes)

Purpose: Establish rapport; ease the participant into talking; help moderator better understand the participant's past and current situation.

1. How many children do you have and what are their ages?

   ……

2. You are here because you have a young child with type 1 diabetes. Tell me about that…

   When was your child diagnosed with T1D?

   ……

3. What have been the biggest challenges for your family in regards to your child's T1D?

   ……

4. Have you been able to get the information you need to understand and care for your child's condition?

   What resources have you used?
   Specifically, what online resources have you used?

   ……

5. What resources or information do you wish you had – either when your child was first diagnosed or now?

   ……

TASKS

Purpose: Learn if users can easily find varying types of information on the site, what they think about the information available on the site, and how to further improve.

For our review today, you will access a website prototype using your phone. A prototype means the website is not finished yet – it's still in development and many of the links and functionality are not hooked up. You may see some gibberish-type text on the site, which just means it's placeholder text and the content is not completed yet. Also, the browser back button does not work on this prototype. So in order to move through the site you will have to use links and/or the site navigation. Other than that, please do whatever you would normally do on a real website.

Show the participant the camera and demonstrate how to hold the phone. Option for wi-fi.

1. **Using your phone's browser, please open this web page, and take a few minutes to explore the site to familiarize yourself with what's available on this site. Do whatever you would do in real life, but remember to think out loud.**
   http://cashdollardesign.com/T1D/Prototype/prototype.html

   Do they read the newly diagnosed overlay? Y N
   Do they select link from the overlay? Y N Link: ……………………………………………………………………………………
   Do they close the overlay w/o interacting with it? Y N

   Observations: ……

If user closes the overlay w/o interacting with it….

2. **So you just closed that window. What was the information on that window about?**
   Feedback: ……

If user links away from the home page….

3. **Please return to the site home page.**
   Path: Logo Home Breadcrumb

   ___ Completed Successfully (Passed = Completed easily without prompting or backtracking)
   ___ Completed with Difficulty (Had Issue = Required prompting or had to backtrack at least once)
   ___ Not Completed or Don't Know (Incomplete = Required prompting and was unsuccessful or gave up)

   Observations: ……

4. **Based on what you've seen so far, what is the purpose of this website?**
   Feedback: ……

5. **Who is this website for?**
   Feedback: ……

6. **Based on what you see on this home page, what type of information can you find on this site?**
   Feedback: ……

7. **Where would you find information about ideas to help you take care of your child with diabetes? For example, how to manage the everyday challenges of raising a child with diabetes.**
   Path: Menu/Problem Solving/+ Your Child with T1D Other: ……………………………………………………………………………

   ___ Completed Successfully (Passed = Completed easily without prompting or backtracking)

___ Completed with Difficulty (Had Issue = Required prompting or had to backtrack at least once)
___ Not Completed or Don't Know (Incomplete = Required prompting and was unsuccessful or gave up)

Observations: ……

8. **Take a moment to explore this page and describe what you're seeing.**

Do they expand the description at top of page? Y N
Do they notice or interact with "all articles" drop-down menu? Y N
Do they scroll all the way down the page to see all articles? Y N
Do they notice/mention ways to sort the articles? Y N

Observations: ……

9. **Whom are the articles written by?**
Health Professionals Parents Others? I don't know

Observations: ……

10. **What do you think about the types of articles and information provided on this page?**
Feedback: ……

11. **Where would you find information to help address how your child's diabetes may be impacting your family? For example, how to talk about diabetes with your other children?**
Path: Menu/Problem Solving/+ Family Other: ……………………………………………………………………………………

___ Completed Successfully (Passed = Completed easily without prompting or backtracking)
___ Completed with Difficulty (Had Issue = Required prompting or had to backtrack at least once)
___ Not Completed or Don't Know (Incomplete = Required prompting and was unsuccessful or gave up)

Observations: ……

12. **Take a moment to explore this page and describe what you're seeing.**

Do they expand the description at top of page? Y N
Do they notice "all articles" drop-down menu? Y N
Do they scroll all the way down the page to see all articles? Y N
Do they notice/mention ways to sort the articles? Y N

Observations: ……

13. **Who are the articles written by?**

Health Professionals Parents Others? I don't know

Observations: ……

14. **What do you think about the types of articles and information provided on this page?**
Feedback: ……

15. **Where would you find information about the latest diabetes research?**
Path: Menu/T1D News & Events   Other: ……………………………………………………………………………………………………

___ *Completed Successfully*   *(Passed = Completed easily without prompting or backtracking)*
___ *Completed with Difficulty*   *(Had Issue = Required prompting or had to backtrack at least once)*
___ *Not Completed or Don't Know*   *(Incomplete = Required prompting and was unsuccessful or gave up)*

Observations: ………

16. **Where would you find information on this site about how to take care of yourself? For example, the importance of prioritizing sleep?**
Path: Menu/Problem Solving/+ Parents   Other: ………………………………………………………………………………………………

___ *Completed Successfully*   *(Passed = Completed easily without prompting or backtracking)*
___ *Completed with Difficulty*   *(Had Issue = Required prompting or had to backtrack at least once)*
___ *Not Completed or Don't Know*   *(Incomplete = Required prompting and was unsuccessful or gave up)*

Observations: ………

17. **Take a moment to explore this page and describe what you're seeing.**

Do they expand the description at top of page?   Y   N
Do they notice "all articles" drop-down menu?   Y   N
Do they scroll all the way down the page to see all articles?   Y   N
Do they notice/mention ways to sort the articles?   Y   N

Observations: ………

18. **What do you think about the types of articles and information provided on this page?**
Feedback: ……

19. **Where would you find information to educate yourself on T1D and its treatment?**
Path: Menu/Problem Solving/+ Learning About T1D   Other: …………………………………………………………………………

___ *Completed Successfully*   *(Passed = Completed easily without prompting or backtracking)*
___ *Completed with Difficulty*   *(Had Issue = Required prompting or had to backtrack at least once)*
___ *Not Completed or Don't Know*   *(Incomplete = Required prompting and was unsuccessful or gave up)*

Observations: ………

20. **Take a moment to explore this page and describe what you're seeing.**

Do they expand the description at top of page?   Y   N
Do they notice "all articles" drop-down menu?   Y   N
Do they scroll all the way down the page to see all articles?   Y   N
Do they notice/mention ways to sort the articles?   Y   N

Observations: ………

21. **What do you think about the types of articles and information provided on this page?**
Feedback: ………

22. **Next, I'd like to get your feedback on an article. Let's imagine that you want to find information specifically about what to do and say when your child with T1D says that they feel different? Where would you find an article about that on this site?**
Path: Menu/Problem Solving/+ Your Child With T1D Other: ………………………………………………………………………………

___ *Completed Successfully* *(Passed = Completed easily without prompting or backtracking)*
___ *Completed with Difficulty* *(Had Issue = Required prompting or had to backtrack at least once)*
___ *Not Completed or Don't Know* *(Incomplete = Required prompting and was unsuccessful or gave up)*

Observations: ……

If user cannot find article, ask them to go back to the home page and link to article from the home page.

23. **Take as much time as you need, and let me know when you're ready to discuss it.**
What do you think about this article?
Feedback: ………

24. **How could you learn more about the author of this article?**
Do they indicate select author's name? Y N
What would you expect to happen when you select the author's name?
Feedback: ………

25. **How could you share this article with someone else?**
Do they select share/email icon? Y N
Feedback: ………

26. **How could you save this article if you wanted to read it again later?**
Do they select bookmark? Y N
Feedback: ………

If user selects bookmark…

27. **Where would you go to find the articles on this site that you have bookmarked?**
Path: Menu/My Account/My Bookmarks Other: ………………………………………………………………………………

___ *Completed Successfully* *(Passed = Completed easily without prompting or backtracking)*
___ *Completed with Difficulty* *(Had Issue = Required prompting or had to backtrack at least once)*
___ *Not Completed or Don't Know* *(Incomplete = Required prompting and was unsuccessful or gave up)*

Observations: ……

28. **Do you ever participate in online diabetes discussion groups with other parents?**
Y N
Why or Why not?: ……

29. **If you wanted to start or join a discussion about diabetes with other parents on this site how would you do that?**
Path: Menu/Parent-to-Parent Forum Other: ………………………………………………………………………………

___ *Completed Successfully* *(Passed = Completed easily without prompting or backtracking)*

___ *Completed with Difficulty* (*Had Issue = Required prompting or had to backtrack at least once*)
___ *Not Completed or Don't Know* (*Incomplete = Required prompting and was unsuccessful or gave up*)

Observations: ………

30. **This page is still under development, but what do you think about this feature?**
Feedback: ………

31. **Are there things you think the site developers should consider when it comes to this type of forum?**
Feedback: ………

32. **If you were to use this website in real life, how likely or unlikely would you be to submit a story or photos about your child with T1D or your family? Using a scale of 1 to 5, with 1 being Very Unlikely and 5 being Very Likely?**
Very Unlikely 1 2 3 4 5 Very Likely

Why or why not?
Feedback: ………

33. **How would you submit a story or photos to this website about your family's experiences?**
Path: Menu/About the New Normal/+ Contact Us Other: ………………………………………………………………………………

___ *Completed Successfully* (*Passed = Completed easily without prompting or backtracking*)
___ *Completed with Difficulty* (*Had Issue = Required prompting or had to backtrack at least once*)
___ *Not Completed or Don't Know* (*Incomplete = Required prompting and was unsuccessful or gave up*)

Observations: ………

34. **Take a moment to explore this page and describe what you're seeing.**

Do they notice the drop-down menu? Y N
Do they notice the different selections? Y N
Do they notice how you upload a file or image? Y N

What do you think about the options available on this page?
Feedback: ………

Please return to the site home page.

35. **I'd like to get your feedback on another article. Please read the article "Our Diagnosis Story" (by Andrea Keglovits) available here on the home page. Take as much time as you need, and let me know when you're ready to discuss it.**

What do you think about this article?
Feedback: ………

36. **What do you think about having articles on the site from parents?**
Feedback: ………

Ask only if user has not yet engaged with the search tool on their own…
37. **How would you find something on this site if you couldn't find it using the site navigation or links?**
Path: Search Icon Other: ……………………………………………………………………………

_ _ _ Completed Successfully (Passed = Completed easily without prompting or backtracking)
_ _ _ Completed with Difficulty (Had Issue = Required prompting or had to backtrack at least once)
_ _ _ Not Completed or Don't Know (Incomplete = Required prompting and was unsuccessful or gave up)

Observations: ……

38. **Whose website is this? (or who is presenting this website, what organization is providing this website?)**
   Feedback: ……

39. **Where would you go if you wanted to better understand how the website was created or the people responsible for it?**
   Path: Menu/About the New Normal or the pages within section Other: ……………………………………………………

_ _ _ Completed Successfully (Passed = Completed easily without prompting or backtracking)
_ _ _ Completed with Difficulty (Had Issue = Required prompting or had to backtrack at least once)
_ _ _ Not Completed or Don't Know (Incomplete = Required prompting and was unsuccessful or gave up)

Observations: ……

Ask if user cannot answer this question and cannot find the About page…

40. **How important is to you to know whose website this is, or who is presenting the information? Using a scale of 1 to 5 where 1 is Not important at all and 5 is very important…**
   Not Important At All 1 2 3 4 5 Very Important

   Why or why not?
   Feedback: ……

   If they answer a 4 or 5…
   How could the site be clearer about whose website this is?
   Feedback: ……

WRAP UP – (5 minutes)

That completes our work with the website prototype. I'd like to ask you a few final questions before we close.

41. **How easy or difficult was it to use the website? Using a scale of 1 to 5, where 1 is Very Difficult and 5 is Very Easy?**
   Very Difficult 1 2 3 4 5 Very Easy

   Why was it difficult or easy?
   Feedback: ……

42. **How would you compare this site to other diabetes information websites you may have used in the past? Would you say it's:**
   ____ Worse
   ____ About the Same
   ____ Better

   Why was it better or worse?
   Feedback: ……

43. **What did you like most about the website?**

……

44. **What could be improved about the website?**

……

45. **Are there any types of information or features that you did not notice on this website that you think should be included in the final product?**

……

46. **Is there any other feedback you'd like to provide about the site?**

……
